# AcalPred: A Sequence-Based Tool for Discriminating between Acidic and Alkaline Enzymes

**DOI:** 10.1371/journal.pone.0075726

**Published:** 2013-10-09

**Authors:** Hao Lin, Wei Chen, Hui Ding

**Affiliations:** 1 Key Laboratory for NeuroInformation of Ministry of Education, Center of Bioinformatics, School of Life Science and Technology, University of Electronic Science and Technology of China, Chengdu, China; 2 Department of Physics, School of Sciences, Center for Genomics and Computational Biology, Hebei United University, Tangshan, China; University of South Florida College of Medicine, United States of America

## Abstract

The structure and activity of enzymes are influenced by pH value of their surroundings. Although many enzymes work well in the pH range from 6 to 8, some specific enzymes have good efficiencies only in acidic (pH<5) or alkaline (pH>9) solution. Studies have demonstrated that the activities of enzymes correlate with their primary sequences. It is crucial to judge enzyme adaptation to acidic or alkaline environment from its amino acid sequence in molecular mechanism clarification and the design of high efficient enzymes. In this study, we developed a sequence-based method to discriminate acidic enzymes from alkaline enzymes. The analysis of variance was used to choose the optimized discriminating features derived from *g*-gap dipeptide compositions. And support vector machine was utilized to establish the prediction model. In the rigorous jackknife cross-validation, the overall accuracy of 96.7% was achieved. The method can correctly predict 96.3% acidic and 97.1% alkaline enzymes. Through the comparison between the proposed method and previous methods, it is demonstrated that the proposed method is more accurate. On the basis of this proposed method, we have built an online web-server called AcalPred which can be freely accessed from the website (http://lin.uestc.edu.cn/server/AcalPred). We believe that the AcalPred will become a powerful tool to study enzyme adaptation to acidic or alkaline environment.

## Introduction

An enzyme is able to multiply the speed of a chemical reaction by lowering the activation energy of participant molecules without any physical or chemical change. Due to high selectivity and catalytic efficiency, enzymes have been widely used in industry, medicine and environment management. Improving catalytic efficiency of enzymes has become the most important task of enzyme engineering. Although rational design and directional evolution can make the designed enzymes work better, environmental conditions also influence their activities. Solubility, temperature and pH value significantly influence enzyme activity [1]. Protein solubility is the basic condition in most biochemical experiments [2]. Enzyme activity increases with temperature rise because the heat enhances the kinetic energy of both substrates and enzymes, which results in more contact between them [3]. Catalytic efficiency is also largely influenced by pH value of their surroundings as the charge of amino acids varies with pH value [4]. In general, an enzyme has an optimum pH. Although most enzymes remain high activity in the pH range between 6 and 8, some specific enzymes work well only in extremely acidic (i.e. pH <5.0) or alkaline (i.e. pH >9.0) conditions. Some acidic and alkaline enzymes derived from acidophiles and alkaliphiles make these organisms survive in high acidic or alkaline conditions [5]. These enzymes also have great potentials in industrial applications. Thus, determination of the favorable pH value of an enzyme is important in academic study and industrial application.

Although the optimized environmental conditions can be obtained by the biochemical experimental approaches, the wet experimental technique is time-consuming and high-cost. Hence, it is highly desirable to develop theoretical methods for predicting appropriate environment of enzymes. The properties in primary sequences of enzymes correlate with their surrounding factors [2], [6], [7]. According to the correlation, machine learning methods have been proposed to predict soluble proteins [8]–[10] and thermophilic proteins [3], [11]–[16] with the information derived from primary sequence. However, few successful cases were reported to predict acidic and alkaline enzymes based on their sequences because it was difficult to collect enough sequence and structure information about acidic and alkaline enzymes [6]. The growing experimental-confirmed proteins in recent years provide a chance to establish bioinformatics methods for accurate discriminating acidic enzymes from alkaline enzymes. Acidic and alkaline enzymes have some particular amino acids [17]. Based on these findings, Zhang et al. [6] presented a random forest model to distinguish acidic enzymes from alkaline enzymes by using sequence and structure information. The model can achieve the overall accuracy of 90.7% in the 10-fold cross-validation. However, the accuracy is still far from satisfaction. Besides, some high homologous sequences in their benchmark datasets result in the overestimation of accuracy. Furthermore, they did not provide a web server so that their method cannot be easily used to obtain desired data by the experimental scientists. Recently, Fan et al. [18] built a free web server called Pred-enzyme to predict acidic and alkaline enzymes. The predictor can achieve an overall accuracy of 94.01% in 10-fold cross-validation. However, their predictor needs gene ontology (GO) information. Our statistical results show that most of proteins have no GO information (<50%). If a query protein has not been annotated in GO database and no homologous can be found in GO database, the prediction with the model is not available.

To overcome these disadvantages, we developed an effective method to discriminate acidic enzymes from alkaline enzymes based on their sequence information alone. A feature selection technique was used to pick out a number of informative features. On the basis of these features, the support vector machine (SVM) was performed to establish prediction model. Jackknife cross-validation was used to evaluate the performance of the proposed method. Prediction results demonstrate that the proposed method is reliable. Based on this method, a free online server called AcalPred was built to provide a useful tool for basic academic study and industrial application of acidic and alkaline enzymes.

## Materials and Methods

### Benchmark Dataset

The original dataset used in this study was obtained from Zhang et al. [6] who extracted the protein annotation information and sequences from enzyme database BRENDA [19] at http://www.brenda-enzymes.info/. In this dataset, only the acidic enzymes with optimal pH below 5.0 and alkaline enzymes with optimal pH above 9.0 were selected. Enzymes with sequence length less than 100 amino acids have been removed. This original dataset contains 105 acidic enzymes and 111 alkaline enzymes. It is well known that high similarity data can lead to erroneous estimation of the performance of the methods. To reduce homologous bias and redundancy, the program PISCES [20] was used to remove those enzymes that have more than 25% pairwise sequence identity to any other. Finally, the benchmark dataset contains 54 acidic enzymes and 68 alkaline enzymes. The 122 enzymes can be freely downloaded from our website (http://lin.uestc.edu.cn/server/AcalPred/data).

### The g-gap Dipeptide Composition

In pattern recognition, one of the key points is to generate a set of informative parameters. It has become a challenge in protein prediction to formulate proteins with an effective mathematical expression for truly reflecting the intrinsic properties of proteins. In the past two decades, various sequence parameters such as amino acid composition (AAC) [3], pseudo amino acid composition (PseAAC) [18] and position-specific scoring matrix (PSSM) [18] have been successfully employed to predict protein structure and function. Because the proximate dipeptide compositions can be used to describe the correlation between two proximate residues, they have been widely applied in protein prediction [21], [22]. However, the intrinsic properties of protein sequences may be deposited in higher tier correlation of residues because of the hydrogen bonding in secondary structure [23], [24]. Thus, we extended the proximate dipeptide composition to the *g*-gap dipeptide composition which can be used to describe the correlation between two residues.

Suppose a protein sequence ***P*** with *L* amino acid residues as follows:
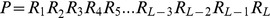
(1)where *R*_1_ represents the amino acid residue at the sequence position 1, *R*_2_ represents the amino acid residue at position 2 and so on. For each *g* of the *g*-gap dipeptide, the feature vector of the protein sequence contains 20×20 = 400 components and can be formulated as:
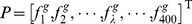
(2)where the symbol **T** denotes the transposition of the vector; 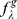 denotes the frequency of the λ-th g-gap dipeptide and is defined as:
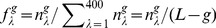
(3)where 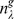 denotes the number of the λ-th g-gap dipeptide. g = 0 indicates the correlation of two proximate residues; g = 1 describes the correlation between two residues with one residue interval; g = 2 indicates the correlation between two residues with the interval of two residues and so forth.

### Feature Selection Technique

Generally, the high dimension vector in feature set would cause the following three problems [25]: one is over-fitting which results in low generalization ability and overestimation of prediction model; another is information redundancy or noise which results in bad prediction accuracy and error description of intrinsic properties; the other is dimension disaster which results in a handicap for the computation or increase of computational time. To overcome these advantages and improve the prediction quality, it is necessary to pick out informative parameters with feature selection techniques to gain deeper insights into the intrinsic properties of protein sequences. Obviously, the best feature combination can be surely achieved by examining the performance of all kinds of feature sets. However, the computation time is so long that we cannot complete it. For economizing run-time and computational resource, a wise strategy is to use algorithm to find the optimal features.

Owing to the development of probability and statistics, some techniques such as principal component analysis (PCA) [26], minimal-redundancy-maximal-relevance (mRMR) [27] and diffusion maps [28] have been presented in sequence analysis and prediction. This study proposed a statistics-based algorithm called the analysis of variance (ANOVA) to score each of the features. The principle of ANOVA is to calculate the ratio (*F* value) of features between groups and within groups for measuring feature variances [21]. Then the *F* value (*F*(*λ*)) of the *λ*-th *g*-gap dipeptide in benchmark dataset is defined by:
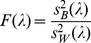
(4)where 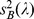 and 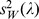 are the sample variance between groups (also called Means Square Between, MSB) and sample variance within groups (also called Mean Square Within, MSW), respectively. They are given by:



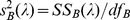
(5)

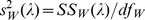
(6)where 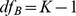 and 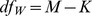 are degrees of freedom for MSB and MSW, respectively. *K* and *M* represent the number of groups and total number of samples, respectively. 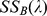 and 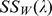 are the sums of squares of the *λ*-th feature between groups and within groups, respectively, which can be calculated by:



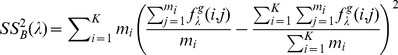
(7)

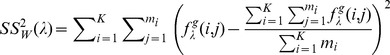
(8)where 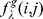 denotes the frequency of the *λ*-th *g*-gap dipeptide of the *j*-th sample in the *i*-th group. The *m_i_* denotes the number of samples in the *i*-th group. Thus we have 
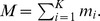


The *F*(*λ*) value in Eq.(4) reveals the correlation between the *λ*-th feature and the group variables. The *F*(*λ*) will become large as the MSB becomes increasingly larger than the MSW. In the absence of differences between groups, the *F*(*λ*) will be near to 1. In other words, the features with a larger *F*(*λ*) indicate that it is a more highly relevant one for the target to be predicted. Hence, the features can be initially ranked according to *F* value in Eq.(4). Subsequently, the incremental feature selection (IFS) is used to determine the optimal number of features. The IFS procedure includes the following steps: starting with one feature with the highest score in the feature set, adding the second feature with the second high score, adding the third feature with the third high score and repeating this process until all candidate features are added. Thus, for each gap *g*, there are 400 feature subsets consisted of 400 ranked *g*-gap dipeptides. Thus, the *t*-th feature subset is composed of *t* ranked *g*-gap dipeptides and can be expressed as:
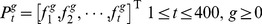
(9)


For 400 feature sets, the prediction accuracy was examined on the benchmark dataset by using jackknife cross-validation. Then we obtained the IFS curve in a 2D Cartesian coordinate system with index *t* as its abscissa (or *X*-coordinate) and the overall accuracy as its ordinate (or *Y*-coordinate). When the *g* was selected from 0 to *g*_0_, there are *g*_0_ IFS curves. With the peaks (or maximum accuracies) of these curves and comparison results of these accuracies, the optimal feature subset with parameters *t*_0_ and *g*_0_ can be obtained and expressed as:
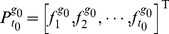
(10)which can provide the maximum accuracy. Then the high-dimensional data can be projected into a low-dimensional space. The final classifier model was built by the optimal feature subset.

### Support Vector Machine

Support vector machine (SVM), as a powerful machine learning method, has been widely and successfully applied in protein bioinformatics [29]–[31]. The basic idea of SVM is to map data of samples into a high dimensional Hilbert space and use kernel function to seek a decision boundary that is able to separate two training data. The decision boundary is a hyperplane which can maximize the margin between the two sets in the feature vector space [32].

In this study, the software LibSVM designed by Lin’s lab was used to implement SVM [33]. In this software, four kinds of kernel functions of linear function, polynomial function, sigmoid function and radial basis function (RBF), can be used to perform prediction. Empirical studies have demonstrated that the RBF outperforms the other three kinds of kernel functions in nonlinear classification. Thus the RBF kernel function was used in the current work. The regularization parameter *C* and the kernel width parameter *γ* were optimized via an optimization procedure according to a grid search approach. In grid research, the search spaces for parameter *C* and *γ* are from 2^15^ to 2^−5^ and from 2^−5^ to 2^−15^ with the steps of 2^−1^ and 2, respectively. The jackknife cross-validation was adopted in this search.

### Performance Assessment

The predictive capability and reliability of the method is estimated by four parameters: sensitivity (*S* *_n_*), specificity (*S* *_p_*), correlation coefficient (*CC*) and overall accuracy (*Ac*) that are defined as follows:
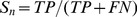
(11)

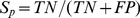
(12)

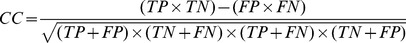
(13)

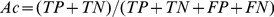
(14)where *TP* denotes the numbers of the correctly recognized alkaline enzymes; *FN* denotes the numbers of the alkaline enzymes recognized as acidic enzymes; *FP* denotes the numbers of the acidic enzymes recognized as alkaline enzymes; *TN* denotes the numbers of correctly recognized acidic enzymes.

## Results and Discussion

In statistical prediction, the following three cross-validation methods are often used to evaluate the performance of a predictor: independent dataset test, subsampling (K-fold cross validation) test, and jackknife test [34], [35]. Among the three cross-validation methods, the jackknife test is the least arbitrary and the most objective because it can yield a unique result for a given benchmark dataset, and hence has been increasingly used by investigators to examine the quality of various predictors. Accordingly, we adopted the jackknife cross-validation in this study to examine the anticipated success rates of the predictor.

### Predictive Accuracy

**Figure 1:**
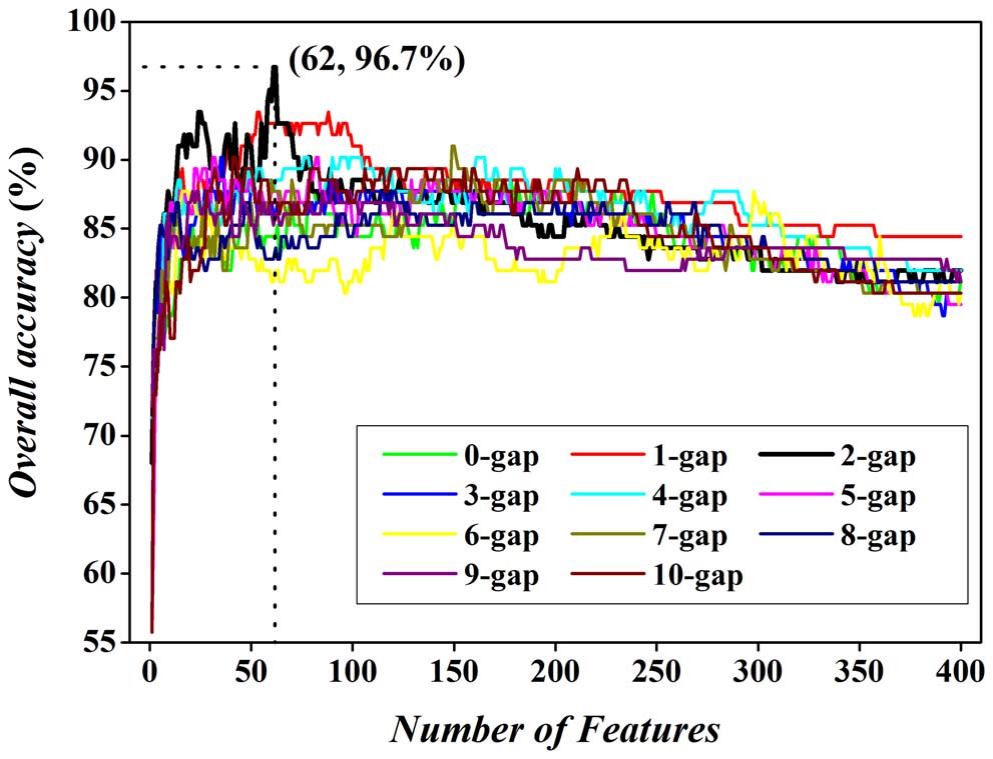
A plot to show the IFS procedure. When the top 62 2-gap dipeptides were used to perform prediction, the overall success rate reached its peak of 96.7%.

The correlation between two arbitrary amino acids with a distance of *g* amino acids can be reflected by the frequencies of the *g*-gap dipeptides (Eq.(3)). For each gap *g*, we must find out the best feature subset which can achieve the best result. Here, taking 2-gap dipeptides as an example, we show the way to achieve the anticipated result. At first, the 400 2-gap dipeptides were ranked according to their *F* values as defined by Eq. (4). The ranked 2-gap dipeptide with a higher *F* value suggests that it is a more highly relevant one for the discrimination between acidic and alkaline enzymes. Subsequently, based on the ranked 2-gap dipeptides, we can build 400 individual predictors for the 400 sub-feature sets by adding the ranked 2-gap dipeptides one by one from higher to lower ranks. It is well known that the sub-feature sets with high *F* value can give more reliable information for classification. However, the number of the selected features is too small to afford enough information, which results in the poor prediction accuracy. For example, the 30^th^ predictor can only produce the overall accuracy of 89.3% in jackknife test. On the contrary, the high dimension sub-feature sets contain enough information. However, the reduction of cluster-tolerant capacity of prediction model will lead to a bad prediction in cross-validation. An example is that the jackknife cross-validated accuracy of 400^th^ predictor is only 82.0%. Therefore, the third step is to investigate the prediction performance for each of the 400 predictors with jackknife cross-validation and then plot the IFS curve. According to the IFS curve shown in Fig. 1, the overall accuracy reached its peak (Ac = 96.7%) when the top ranked 62 2-gap dipeptides were used. These dipeptides have the *F* score more than 6.39 (*P*-value<0.0128). The successful prediction rates were 96.3% and 97.1% for acidic and alkaline enzymes, respectively.

It is necessary to investigate whether other *g*-gap sub-feature sets can obtain higher accuracies or not. We changed the *g* from 0 to 10 and repeated the feature selection process to find the maximum accuracy of each *g*-gap dipeptides. For the convenience of observation and comparison, eleven IFS curves (*g* varying from 0 to 10) were plotted in Fig. 1. These results indicate that the sub-feature set with parameters *t*_0_ = 62 and *g*_0_ = 2 is the best one among the 4400 (400×11) optimized feature sets. The area under receiver operating characteristic (ROC) curve (AUC) achieves 0.956 in the jackknife cross-validation.

**Figure 2:**
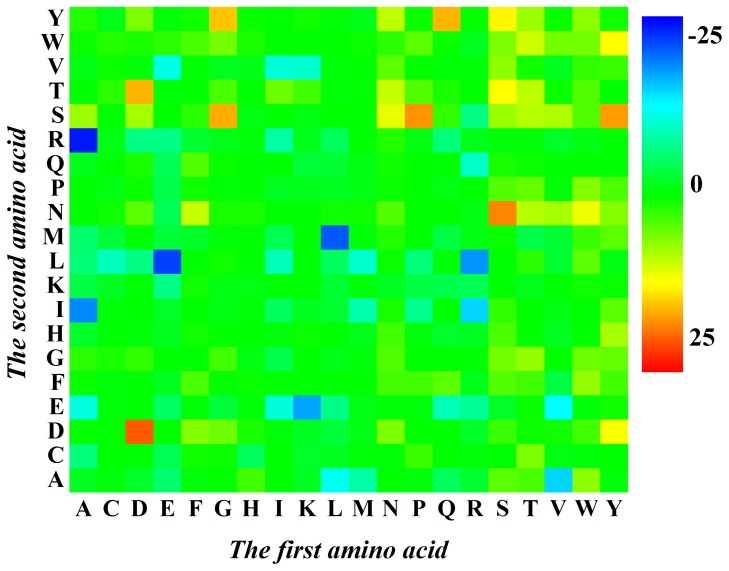
A chromaticity diagram for the *F* values of 400 2-gap dipeptides. The blue boxes were positively correlated with acidic enzymes, while the red boxes were positively correlated with alkaline enzymes.

To provide an overall view, the distribution for the *F* values of the 400 2-gap dipeptides and their roles for the prediction model were given in Fig. 2. The features in blue boxes were positively correlated with acidic enzymes, while those in red boxes were positively correlated with alkaline enzymes. As shown in Fig.2, Arg (R), Leu (L) and Ile (I) are preferred in acidic enzymes and Asp (D), Tyr (Y), Ser (S) and Thr (T) are preferred in alkaline enzymes. The Arg is a basic amino acid with the largest Isoelectric point (10.76) among 20 types of amino acids, whereas the Asp is an acidic amino acid with the smallest Isoelectric point (2.98) among 20 types of amino acids. The pH environment has a major effect on ionic binding, which is essential for enzyme activation and chemical reactions. The Arg δ-guanido moiety can provide more surface area for charged interactions and more easily maintains ion pairs and a net positive charge at elevated pH [36]. Therefore, the reason that acidic or alkaline enzymes contain many basic or acidic amino acids is that they need such specific residues to neutralize with extremely acidic (pH <5.0) or alkaline (pH >9.0) surroundings for executing enzymes’ activities.

For demonstrating the prediction capability of the proposed model, we built an independent dataset which contained 20 acidic and 20 alkaline enzymes. These sequences derived from BRENDA [19] can be freely downloaded from our website. The sequence identity between training benchmark dataset and independent set is less 40%. Our model can correctly identify the 19 acidic and 20 alkaline enzymes. Furthermore, we investigated the accuracy of our method on another independent constructed by Fan et al. [18]. Results showed that 17 acidic and 16 alkaline enzymes could be correctly predicted when optimized cutoff was selected.

### Comparison with Other Methods

To further demonstrate the performance of the proposed method, it is necessary to compare it with other existing methods. However, it is not objective and strictly to directly compare the results due to different benchmark datasets. Therefore, we repeated the process of feature selection and prediction on the original dataset (105 acidic and 111 alkaline enzymes). It should be noted that the results reported by Zhang et al. [6] and Fan et al. [18] were derived by 10-fold cross-validation test. As elucidated by Chou [35], their test can not provide unique result. For the current case, the benchmark data set contains 105 acidic and 111 alkaline enzymes. According to the Equations 28 and 29 in [35], [37], if one tenth samples are selected from each of the two subsets for conducting the 10-fold cross-validation, the number of possible combinations will be more than 10^29^, which is too large to be completed. Therefore, in previous studies [6], [18], one of 10^29^ possible combinations is randomly picked out to perform the 10-fold cross-validation. To make the comparison between our method and their methods with the same test method, we also randomly picked one of the possible combinations from the same benchmark data set to perform the 10-fold cross-validation test and the compared results were recorded in Table 1.

**Table 1:** Comparing the performance of the proposed method with other existing methods.

	*Sn*(%)	*Sp*(%)	*CC*	*Ac*(%)	*AUC*(%)
**Our method**	**94.6**	**94.3**	**0.89**	**94.4**	**0.975**
Zhang’s method	88.6	92.8	0.82	90.7	0.958
Fan’s method	92.4	95.5	0.88	94.0	0.961

According to Table 1, when the top 81 1-gap dipeptides are used, our method can achieve the maximum accuracy of 94.4% with the AUC of 0.975 in 10-fold cross-validation, which is higher than the maximum accuracy obtained with other methods. Although the *Sp* obtained by our method is not the best, the *Sn*, *CC* and *Ac* are dramatically better than those of other methods, suggesting that the proposed method outperforms other published methods.

We noticed that Zhang et al. [6] and Fan et al. [18] also achieved encouraging results. Zhang’s [6] proposed to use secondary structure amino acid composition as inputting parameters. This kind of information derived from software Predator program. It should be noted that the accuracy of Predator program was only about 75%. If the secondary structure of a protein chain is not correctly predicted, it will provide wrong information for further acidic/alkaline enzyme description. This is the possible reason that the Zhang’s models can not obtain higher accuracies with the predicted secondary structural feature. The parameter sets of Fan et al. [18] model include average chemical shift (acACS) information, Go information and evolutionary (PSSM) information. In fact, their novel feature acACS can only achieve the overall accuracy of 85.7%. Go and PSSM information play an important role in their model construction. It is well known that some proteins don’t have the Go annotation. We investigated the number of proteins in Uniprot and found that less than 50% proteins have GO information. Thus, their model can not provide any information for the protein that has not been annotated in GO database. Moreover, the PSSM information also has shortcomings. The generation of PSSM of a protein depends largely on the searching dataset. If no homologous sequence is found in the searching dataset, the PSSM will not give exact description, thus leading to wrong prediction. With primary sequence information, our model can obtain such high accuracy, suggesting that the proposed model is more neat free and efficient.

### Web-Server Guide

For the convenience of the vast majority of experimental scientists, we built a free web server called AcalPred to discriminate acidic enzymes from alkaline enzymes. Below, let us give a step-by-step guide on how to use the AcalPred web server. Then experimental scientists may get the desired results without the complicated mathematic equations. The detailed steps are provided as follows:

#### Step 1

**Figure 3:**
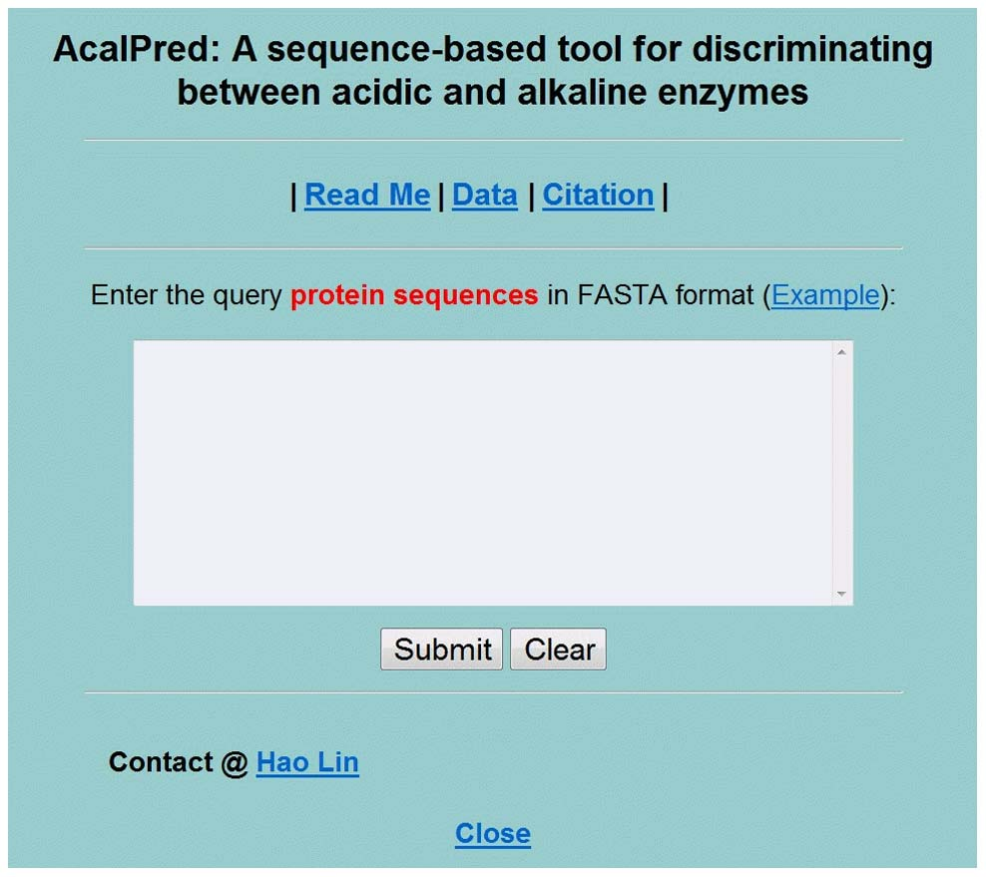
A semi-screenshot to show the top page of the AcalPred web-server. Its website address is at http://lin.uestc.edu.cn/server/AcalPred.

Open the web server at http://lin.uestc.edu.cn/server/AcalPred and you will see the homepage of AcalPred on your computer screen, as shown in Fig. 3. Click on the Read Me button to see a brief introduction about the predictor and the caveat. Users may click on the Data button to download the training set and test set. By clicking on the Citation button, users may find the relevant papers on the detailed development and algorithm of AcalPred.

#### Step 2

Input or copy/paste the query protein sequence that you want to predict into the input text area at the center of Fig. 3. The input sequence should be in the FASTA format. A sequence in FASTA format consists of a single initial line beginning with a greater-than symbol (‘>’) in the first column, followed by lines of sequence data. The words right after the ‘>’ symbol in the single initial line are optional and only used for the purpose of identification and description. All lines should be no longer than 120 characters and usually do not exceed 80 characters. The sequence ends if another line starting with a ‘>’, which indicates the start of another sequence. Example sequences in FASTA format can be seen by clicking on the Example button right above the input box.

#### Step 3

Click on the Submit button to see the predicted result. The probabilities belonging to two classes will be given in the second and third columns. The first column gives the prediction type with prediction probability is above 0.5. For example, if you use the query protein sequences in the Example window as the input, after clicking the Submit button, you will see the following contexts on your screen: the outcome for the first query sample is ‘acidic enzyme’ because the prediction probabilities of acidic enzyme and alkaline enzyme are respectively 0.903627 and 0.096373; the outcome for the second query sample is ‘alkaline enzyme’ because the prediction probabilities of acidic enzyme and alkaline enzyme are 0.074938 and 0.925062, respectively.

## Conclusion

In this work, we developed a promising method to discriminate acidic enzymes from alkaline enzymes. The ANOVA-based feature selection technique was utilized to optimize dipeptide compositions for improving the prediction capability of model. An overall accuracy of 96% was achieved, demonstrating that the proposed model is a powerful tool for the study of enzymes in the adaptation to acidic or alkaline environment. For the convenience of experimental scientists, a free web server AcalPred was built to implement the prediction. A friendly guide was given to describe the way to use the AcalPred web server. We believe that the predictor will be helpful for wet lab scientists who focus on enzyme activity.
